# Reactivation of Q fever: case report of osteoarticular infection developing at the site of a soft tissue injury

**DOI:** 10.1099/acmi.0.000296

**Published:** 2021-12-10

**Authors:** Genevieve L. McKew, Thomas Gottlieb

**Affiliations:** ^1^​ Concord Repatriation and General Hospital, Concord, New South Wales, Australia; ^2^​ University of Sydney, New South Wales, Australia

**Keywords:** Q fever, *Coxiella burnetii*, osteoarticular infection, reactivation

## Abstract

*
Coxiella burnetii *, the causative agent of Q fever, is known to cause acute and persistent infection, but reactivation of infection is rarely reported. This case demonstrates reactivation of a distant, untreated Q fever infection after a relatively innocuous soft tissue injury in an adjacent joint without pre-existing pathology. A 52-year-old male abbatoir worker sustained an adductor muscle tear in a workplace injury. He was unable to walk thereafter, and developed a chronic, progressive, destructive septic arthritis of the adjacent hip with surrounding osteomyelitis of the femur and acetabulum. He had evidence of prior Q fever infection, with a positive skin test and serology 15 years beforehand. He was diagnosed with chronic osteoarticular Q fever on the basis of markedly elevated phase I antibodies, and symptomatic and serological response to prolonged antibiotic treatment with doxycycline and hydroxychloroquine. He required a two-stage hip arthroplasty. This case illustrates reactivation of latent *C. burnetii* infection at the site of a soft tissue injury. Clinicians need to be aware of this possibility in patients with previous Q fever infection, and in the setting of undiagnosed osteoarticular pathology following soft tissue injury.

## Introduction

Osteomyelitis and septic arthritis are rare manifestations of Q fever. The causative agent is *Coxiella burnetii,* a phagolysosome-dwelling organism that can persist within the host [1, 2]. Between one and five per cent of patients with *C. burnetii* develop symptomatic persistent focalized infection [3], more commonly endocarditis than bone infection. Some patients exhibit persistent elevations in phase I antibody titres, without clinical manifestations or ongoing exogenous exposure to the antigen [4]. Clinical reactivation of Q fever is rare, with limited reports in the setting of parturition, cardiac and orthopaedic surgery. This case illustrates a previously unreported manifestation of Q fever in humans: reactivation of latent infection following a soft tissue injury.

## Case report

A 52-year-old abbatoir employee from northern New South Wales, Australia, injured his right thigh while attempting to stop bovine intestines falling with his foot in November 2011. He had sudden onset of pain in the adductor insertion, radiating down the medial thigh. Over the next week, pain developed in the buttock and lateral thigh to the knee. Pain and swelling continued over the next few months.

Ultrasound 10 days post-injury demonstrated a tear of the adductor magnus, and plain X-ray was normal. Magnetic resonance imaging 2 months later showed extensive marrow oedema in the femoral head and neck, acetabulum and adjacent hemi-pelvis, with partial collapse of the femoral head, oedema of the gluteal and adductor muscles, and a tear in obturator externus. There was hip synovitis and fluid. C-reactive protein was 98.9 mg l^−1^.

Subsequent X-rays demonstrated a progressive, destructive process in the femur and acetabulum. Bone scan demonstrated avid focal tracer uptake in the right femoral head and neck. Thirteen months post-injury, the Q fever phase I IgG and total antibody titre was >3200 by immunofluorescence (normal <25), consistent with chronic Q fever. At age 40 at pre-immunization screening, the patient’s Q fever skin test had been positive, with an induration diameter of 15 mm, while the Q fever IgG enzyme immunoassay index was 1.4 (<1.0) and the phase 2 complement fixation titre was 2.5 (8–32). After that time, he had not taken antibiotics that are active against Q fever.

Seventeen months post-injury, he attended the Infectious Diseases service. The hip was mildly tender, in fixed flexion, with severely limited passive movements, and active movements limited by pain, with non-pitting oedema of the leg. There was quadriceps wasting with no muscle tenderness. He commenced doxycycline and hydroxychloroquine. Histopathology from synovial core biopsy demonstrated non-granulomatous chronic inflammation. Bacterial, fungal, mycobacterial and *C. burnetii* culture and PCR were negative after two doses of doxycycline. Cell culture and PCR were processed at the Australian Rickettsial Reference Laboratory (Geelong, Australia). QuantiFERON-TB Gold and brucellosis serology were negative. Echocardiography was normal.

**Fig. 1.**
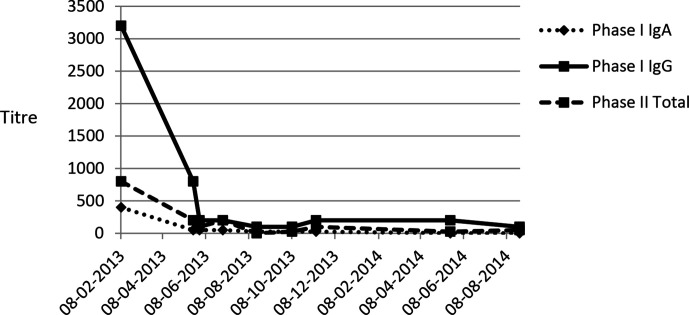
Q fever antibody titres by immunofluorescence. Doxycycline treatment was started in early April 2013.

Pain and mobility were much improved 7 weeks later, at 19 months post-injury. The fixed flexion deformity resolved, with nearly normal range of hip motion. C-reactive protein levels had returned to normal and phase I Q fever antibody titres were much reduced (Fig. 1).

The patient underwent a successful two-stage total hip arthroplasty in February 2014 at 27 months post-injury, taking doxycycline throughout. Standard and *C. burnetii* joint tissue culture and PCR were negative.

The patient received 36 months of doxycycline and hydroxychloroquine, with no relapse in symptoms or radiological change 18 months post-treatment cessation, despite transient elevations in Q fever antibody titres during treatment.

## Discussion

The patient’s illness was consistent with a reactivation of latent Q fever causing septic arthritis and osteomyelitis due to injury, based on serologically confirmed distant Q fever infection, recent illness, sequential imaging, high-titre phase I Q fever serology, and rapid clinical and serological response to treatment.

There are 36 reported cases of osteoarticular infection in adults [5, 6]. Osteomyelitis usually affects the long bones, or the lumbar vertebrae, due to contiguous spread from aortitis. In children, there is a distinct entity of chronic, recurrent multi-site osteomyelitis, which responds to immunosuppressive therapy and has poor response to antibiotics [7, 8].

Reactivation of Q fever is not the typical paradigm of human disease, although it occurs in animals during gestation [9] or under experimental immunosuppression [10]. Isolation of *C. burnetii* has been documented after resolution of acute infection: after a woman with laboratory-acquired infection was treated, *C. burnetii* was isolated from placenta and milk 6 months later; and *C. burnetii* was isolated from the placentas of four women who had had acute Q fever 6 months to 3 years beforehand [11]. This illustrates the biological plausibility of reactivation, demonstrating that *C. burnetii* can persist in a viable state after acute infection. However, definite clinical reactivation in adults has rarely been reported. One case report describes a febrile illness post-aortic valve replacement, evolving into frank Q fever endocarditis, representing quiescent infection with clinical reactivation after surgical tissue injury [12].

Serological reactivation occurred in 7 of 42 patients with pre-existing *C. burnetii* antibodies post-cardiac surgery, all with valve replacements [9]. A case of Q fever endocarditis described an interval of 7 years between acute Q fever and clinical onset, but this could possibly represent subclinical, rather than latent, infection [13]. Four of eight reported cases of Q fever prosthetic joint infection developed after revision surgery [5, 6, 14–16].

This case of apparent reactivation of latent *C. burnetii* infection in injured, previously healthy tissue represents a new paradigm in Q fever. This principle of locus minoris resistentiae has been reported in other infections [17]. Clinicians need to be aware of this possibility in patients with previous Q fever infection, and in patients with undiagnosed osteoarticular pathology following injury.
